# A Paleolatitude Calculator for Paleoclimate Studies

**DOI:** 10.1371/journal.pone.0126946

**Published:** 2015-06-10

**Authors:** Douwe J. J. van Hinsbergen, Lennart V. de Groot, Sebastiaan J. van Schaik, Wim Spakman, Peter K. Bijl, Appy Sluijs, Cor G. Langereis, Henk Brinkhuis

**Affiliations:** 1 Department of Earth Sciences, Utrecht University, Utrecht, The Netherlands; 2 University of Oxford e-Research Centre, Oxford, United Kingdom; 3 Center of Earth Evolution and Dynamics (CEED), University of Oslo, Oslo, Norway; 4 Royal Netherlands Institute for Sea Research (NIOZ), Den Burg, The Netherlands; Wesleyan University, UNITED STATES

## Abstract

Realistic appraisal of paleoclimatic information obtained from a particular location requires accurate knowledge of its paleolatitude defined relative to the Earth’s spin-axis. This is crucial to, among others, correctly assess the amount of solar energy received at a location at the moment of sediment deposition. The paleolatitude of an arbitrary location can in principle be reconstructed from tectonic plate reconstructions that (1) restore the relative motions between plates based on (marine) magnetic anomalies, and (2) reconstruct all plates relative to the spin axis using a paleomagnetic reference frame based on a global apparent polar wander path. Whereas many studies do employ high-quality relative plate reconstructions, the necessity of using a paleomagnetic reference frame for climate studies rather than a mantle reference frame appears under-appreciated. In this paper, we briefly summarize the theory of plate tectonic reconstructions and their reference frames tailored towards applications of paleoclimate reconstruction, and show that using a mantle reference frame, which defines plate positions relative to the mantle, instead of a paleomagnetic reference frame may introduce errors in paleolatitude of more than 15° (>1500 km). This is because mantle reference frames cannot constrain, or are specifically corrected for the effects of true polar wander. We used the latest, state-of-the-art plate reconstructions to build a global plate circuit, and developed an online, user-friendly paleolatitude calculator for the last 200 million years by placing this plate circuit in three widely used global apparent polar wander paths. As a novelty, this calculator adds error bars to paleolatitude estimates that can be incorporated in climate modeling. The calculator is available at www.paleolatitude.org. We illustrate the use of the paleolatitude calculator by showing how an apparent wide spread in Eocene sea surface temperatures of southern high latitudes may be in part explained by a much wider paleolatitudinal distribution of sites than previously assumed.

## Introduction

In the last decade, paleoclimatology has been amongst the most rapidly developing research fields within the Earth Sciences. Crucial information that can be derived from geological records include the relationships between atmospheric chemical composition and global temperature, meridional temperature gradients, and regional and global sea level change, as well as the response of the global exogenic system to perturbations in ocean acidity and oxygenation [1]. Such studies ultimately aim at improving projections of future climate and ecosystem changes resulting from human emissions of carbon and nutrients.

A crucial aspect of paleoclimate reconstructions based on geological proxy data is a correct representation of paleogeography, notably continent-ocean configuration and latitude. Continent-ocean configuration partially determines the distribution of energy across the Earth’s surface via ocean and atmosphere circulation. To accurately reconstruct past climate change, it is therefore essential to accurately constrain the paleogeographical position of a geological record, sampled at a drill site or an exposed sedimentary section, at the moment of deposition. For instance, a measurable/reconstructable (paleo-)climate parameter that provides key information to compare with theory (i.e., climate models) is the meridional temperature gradient. Many of such paleotemperature gradients have been reconstructed, e.g., for the Eocene [2–5]. However, for proper interpretation and comparison to numerical model predictions, the precise position, paleolatitude, and geography of the study sites, as well as the uncertainty on such numbers, must be optimally constrained to assess e.g., solar insolation and its position relative to expected ocean and atmosphere circulation patterns that potentially cause regional variations.

To incorporate the effect of plate tectonic changes, the relative motions of plates are reconstructed using marine magnetic anomalies and fracture zones of the ocean floor. Widely used reconstructions in the paleoclimate community are for instance those of Hay and colleagues [6], Scotese [7], and Müller and colleagues [8]. Using these reconstructions, so-called relative plate motion chains [9] are built that incorporate the relative motions of present and former plates that are or were bounded by mid-ocean ridges.

Such relative plate motion chains, which may be closed into a plate circuit, may be a sufficient reference frame to study the kinematic evolution of a destructive plate boundary, such as the India-Asia plate boundary in the Himalaya. For many other studies, however, it is key to determine the position of the plate circuit relative to the Earth’s spin axis, or to the mantle. To this end, ‘absolute’ reference frames have been developed. Paleoclimate studies require knowledge of the location of a studied sedimentary archive during its deposition relative to the Earth’s spin axis, as this determined its position relative to the sun, and thus its climate.

Below, we illustrate that reference frames reconstructing plates relative to the mantle can considerably differ from frames reconstructing plates relative to the spin axis (perhaps as much as 15° (i.e. ~1650 km) in the Early Cenozoic [10] and more than 20° (>2200 km) in the Mesozoic [11]) due to a process known as ‘true polar wander’. These differences may become important at critical time intervals and specific locations on Earth, for instance close to 60° paleolatitude, where even slight differences in paleolatitude determine whether a given site was in easterly versus westerly winds [12] and it is thus essential to use a reference frame relative to the Earth’s spin axis.

The notion, and importance of using the appropriate reference frame for paleoclimate studies, however, seems somewhat underappreciated in the literature. Most paleoclimate studies do not specify the reference frame that is used to determine the paleolatitude. In more recent literature (e.g., [3]), reference is frequently made to freely online available Gplates reconstruction software [13]. This software package comes with a state-of-the-art relative global plate reconstruction [14], and as default a mantle reference frame [15], which is appropriate for geodynamic problems, but, as we will explain, not for paleoclimate studies. As a consequence, while the effects of the obliquity of the Earth axis that correspond to variations of ~2° are carefully taken into account, the potential uncertainty introduced by misplacement of a study site due to an inappropriate reference frame may be one order of magnitude larger for paleoclimate studies going far back into geological time.

In this paper, we summarize the procedures underlying plate tectonic reconstructions and reference frame generation, and identify which reference frames should be used for various Earth scientific problems. In addition, we describe and provide an online tool (available at www.paleolatitude.org) that we developed to determine the paleolatitude within all major plates and plate fragments, allowing a user to determine the paleolatitude and associated error bars relative to the Earth’s spin axis, tailor-made for paleoclimate analyses of Jurassic and younger times. Finally, we will show a case study in Cenozoic global temperature reconstructions to illustrate the importance of using the appropriate reference frame, and the use of the provided paleolatitude calculator.

## Plate Kinematic Reconstructions and Reference Frames

Paleoclimate studies require constraining the paleolatitude at which sediments were deposited relative to the Earth’s orbit around the sun, to determine paleoclimatic constraints such as the angle of solar insolation. This requires constraints on the obliquity of the Earth’s spin axis, and a reference frame that places the plates relative to that spin axis. As we will explain in this section, only paleomagnetic data can provide this information whereas mantle reference frames cannot because of a process known as ‘true polar wander’. To this end, we will briefly explain how global plate reconstructions and reference frames are built, why different reference frames exist, and how the choice of the appropriate reference frame is crucial for application to a paleoclimate (or geodynamics) study.

### Paleomagnetic observations and paleogeography

**Fig 1:**
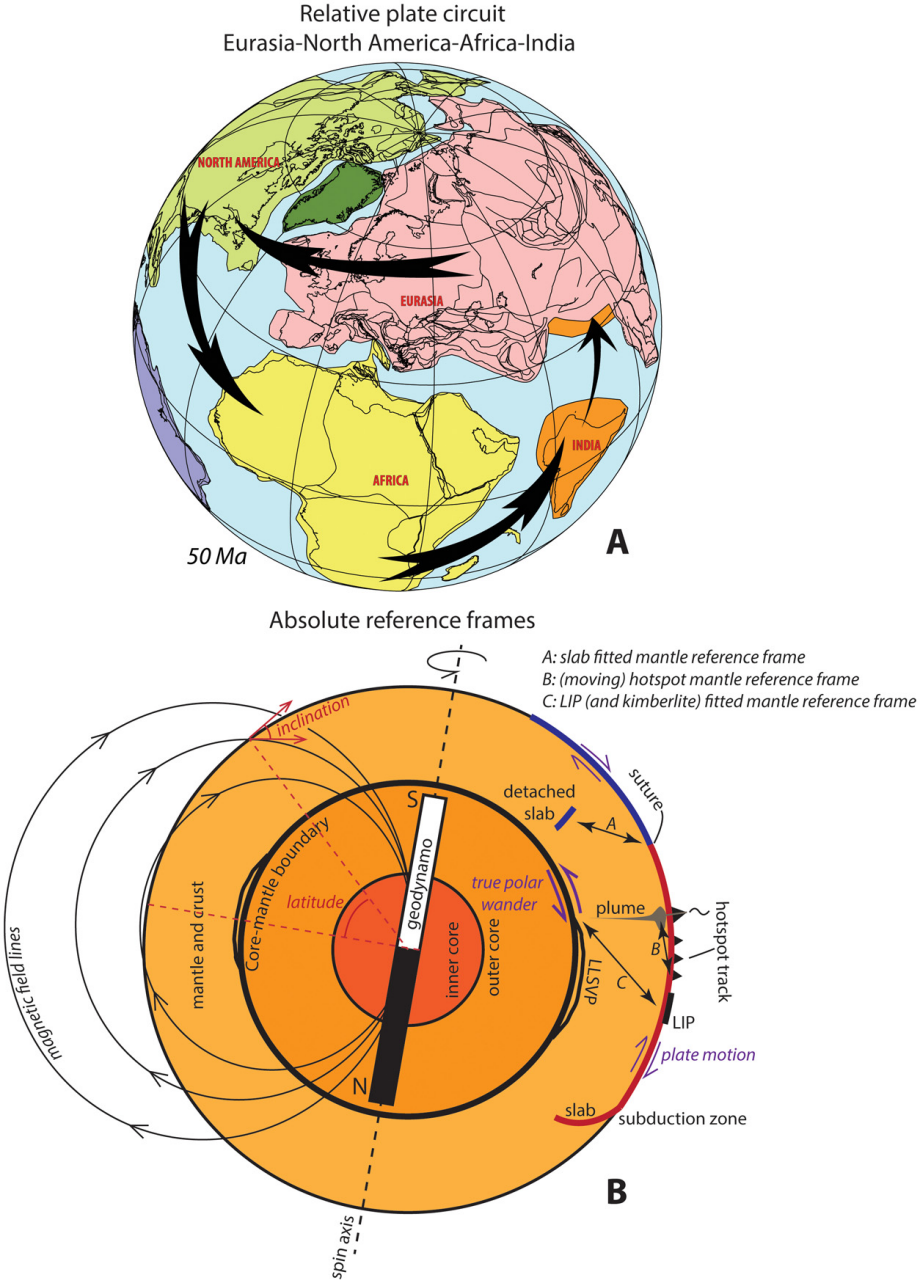
(A) Example of a plate circuit. The motion of India versus Eurasia cannot be directly constrained since these plates are bounded by a destructive plate boundary (trench). Relative motions between these plates can be reconstructed by restoring the opening history of the North Atlantic ocean between Eurasia and North America, the Central Atlantic Ocean between Africa and North America, and the Indian Ocean between India and Africa. With the relative positions of all these plates known through time, a paleomagnetic pole of one of these plates can be used to constrain all of these plates relative to the geodynamo. (B) schematic outline of plate and mantle motions and reference frames. Plates move relative to the mantle (plate tectonics), and plates and mantle together can undergo phases of motion relative to the liquid outer core (true polar wander). Both processes lead to motion of a rock record relative to the Earth’s spin axis, and hence both influence the angle of insolation that is relevant for paleoclimate study. Mantle reference frames *A-C* (see text for explanation of these frames) can only reconstruct plate motion relative to the mantle, but cannot reconstruct true polar wander. These frames are therefore used for analysis of geodynamics, but should not be used for paleoclimate studies. Instead, a paleomagnetic reference frame should be used. On geological timescales, the geodynamo coincides with the Earth’s spin axis. The orientation of the paleomagnetic field in a rock can be used to restore a rock record into its original paleolatitude relative to the spin axis.

The geodynamo that generates the Earth’s magnetic field results from convection in the outer core [16]. On time scales larger than ~10 kyr this dynamo can be considered a dipole that aligns with the Earth’s spin axis [9,16–21]. In an ideal dipole field, the orientation of magnetic field lines is a function of latitude only. In paleomagnetism, the paleomagnetic unit vector tangential to the local magnetic field line is normally decomposed into two directions: the declination, which is the direction of the horizontal component relative to geographical North (spin axis), and the inclination, which is the angle between the vertical component and the horizontal. An inclination is positive when it plunges downward. The inclination I is a function of latitude λ (Fig 1) following the dipole equation:

tanI=2tanλ (1)

Small deviations on the order of several degrees between the magnetic north pole and the spin axis may derive from non-dipole field components, which are generally believed to be <5% of the dipole field components [22]. In addition, on timescales smaller than ~10kyr, the geodynamo undergoes secular variation that may deflect the magnetic pole by tens of degrees from the spin axis. Paleomagnetists therefore carefully sample the rock record to average out this short-term variation, to a precision of a few degrees (i.e. several hundreds of kilometers in paleolatitude), following clear quality criteria [23]. A successful paleomagnetic mean direction plus its error margins are therefore calculated from a distribution of Virtual Geomagnetic Poles (VGPs) that represents paleosecular variation over a sufficient amount of geological time. This VGP distribution is statistically represented by a 95% confidence limit, which is circular (cone-shaped) at the mean VGP of paleopole, expressed by the term ‘A95’ [24]. As a consequence, the distribution of paleomagnetic directions is ellipse-shaped, with Gaussian distributed errors in declination (Δ*D*_x_) and inclination (Δ*I*_x_) [17]. The recorded inclination I ± Δ*I*_x_ thus quantifies the paleolatitude and its error margins. Note that the dipole equation causes the errors in paleolatitude to be asymmetrical. In addition, the recorded declination determines the vertical-axis rotation component of the sampled rock sequence and constrains the total tectonic rotation that occurred since the time of deposition.

If the studied rocks have (latitudinally) moved or rotated after deposition because of plate tectonic motion and/or true polar wander, the recorded paleomagnetic direction will differ from the magnetic direction at the present position. For these rocks, the recorded magnetic paleopoles—in modern geographic coordinates—appear to have ‘wandered’ from some past position to the current geographical North Pole. This ‘apparent polar wander path’ (APWP) forms the basis for the construction of paleomagnetic reference frames for plate motion [25].

### Paleomagnetic reference frame

For times prior to Pangea, i.e. from Paleozoic times and older, apparent polar wander paths have been constructed from paleomagnetic data from individual continents and continental fragments that were separated by oceans that have now entirely been subducted (e.g., [26]). For times since the break-up of Pangea, however, paleomagnetic data from all continents can be used together to determine the motion of plates relative to the Earth’s spin axis, through the construction of a Global Apparent Polar Wander Path (GAPWaP) [20].

Marine magnetic anomalies on the modern ocean floor in combination with transform faults and fracture zones allow for the reconstruction of the relative motions between most modern continents since the break-up of Pangea. Vine and Matthews [27] recognized that magnetic anomalies are mirrored in, and result from, ocean spreading at mid-ocean ridges while recording magnetic polarity reversals. The ages of these magnetic polarity reversals are well determined and summarized in the geomagnetic polarity timescale [28,29]. Thus, the amount and rate of sea floor spreading through time can be quantified and the relative motions of all modern and former plates that are bounded by an active or extinct mid-ocean ridge have been reconstructed (expressed as rotations over Euler poles [30,31]) forming relative plate motion chains [9] (Fig 1A). Typical error bars associated with magnetic anomaly and transform-fracture zone reconstructions are on the order of tens of kilometers only [32], an order of magnitude smaller than typical errors associated with paleomagnetic poles.

When relative motions between plates in a plate motion chain are known through ocean basin reconstructions, a paleomagnetic pole from one of these plates is sufficient to constrain the position of the other plates in the chain relative to the geodynamo. This way, all paleomagnetic poles from the stable continents, and in a few cases from islands built on ocean floor, can be calculated into one common coordinate frame (typically attached to South Africa [9,20]). A global apparent polar wander path (GAPWaP) can then be constructed by averaging all paleomagnetic poles of chosen age bins, which gives associated A95 confidence regions. The latest of these was published by Torsvik and colleagues [20], and we also provide results from earlier versions published by Besse and Courtillot [18,33], which is broadly similar, and Kent and Irving [34], which differs particularly in the Jurassic. The GAPWaP can be used to determine the paleolatitude and total vertical axis rotation of any site on Earth relative to the dipole axis (as proxy for the spin axis) provided that this site is located on a plate that can be connected through a plate motion chain to South Africa. Paleomagnetic data, however, do not constrain paleolongitude: paleolongitude relative to the Earth’s spin axis is irrelevant for climate studies, as it only determines when the sun comes up and goes down in a 24 hour cycle. When portraying paleogeographical maps using a paleomagnetic reference frame, absolute longitude is therefore not quantified, only relative longitudinal positions are shown, following from the global plate circuit [9].

### Hotspot reference frames

To study the interaction of the Earth’s mantle with crust and surface evolution, the geodynamic community has developed so-called mantle reference frames, to analyze e.g., the driving mechanism of plate tectonics, or dynamic surface topography.

Mantle reference frames are developed by linking structures that are presumed to be more or less stationary in the ambient mantle relative to their geological expressions in the rock record. The best known of these are hotspot reference frames. Hotspot tracks, linear chains of intraplate volcanic centers with a regular age progression, are interpreted to reflect plate motion over a stable magma source [35] likely sourced from mantle plumes [36]. The first hotspot reference frames that attempted to place plate motion chains relative to the mantle, assumed hotspot fixity in the upper mantle [25,37]. In the light of evidence that hotspots may actually slowly move relative to one another as a result of plume deflection due to upper mantle convection [38–40], more recently moving hotspot reference frames were developed that correct for this relative plume motion. This was first established for the Indo-Atlantic Realm [15], and recently for the first time for a larger plate motion chain integrating the Indo-Atlantic and Pacific realms [10]. Hotspot reference frames go back to the mid-Cretaceous (100–120 Ma), prior to which time there are insufficient hotspot tracks left due to subduction to carry out a meaningful analysis. Hotspot reference frames thus constrain the paleolatitude and paleolongitude of all plates in the plate motion chain relative to the mantle.

### True Polar Wander

It turns out that hotspot reference frames and paleomagnetic reference frames predict different paleolatitudinal and rotational motions of the global plate motion chain over extended periods of time (10^7^ Myr). Doubrovine and colleagues [10], for instance, calculated that the ‘misfit’ in predicted pole position between their global moving hotspot reference frame and the GAPWaP of Torsvik and colleagues [20] may be as much as ~15° in early Cenozoic time. These differences are physically explained by a uniform rotation of the mantle and crust relative to the geodynamo (spin axis) along the core-mantle boundary. This is caused by the ongoing redistribution of mass by mantle convection and crustal deformation continuously perturbing the Earth’s moments of inertia. Such a uniform rotation thus rotates the mantle and crust relative to the spin axis [41,42], a process known as *true polar wander* (Fig 1B). Because a hotspot reference frame constrains motions of plates relative to the mantle, the common motion of plates and mantle together relative to the core (i.e. true polar wander) goes unnoticed in such frames. As a result, paleolatitudes predicted by a mantle reference frame may differ from paleolatitudes predicted by a paleomagnetic reference frame. The magnitude and timing of true polar wander events may be calculated from the difference in pole positions predicted by a hotspot reference frame and a paleomagnetic reference frame (e.g., [10,18]). Alternative procedures have been developed to calculate true polar wander from paleomagnetic data alone [11].

The possibility to correct a paleomagnetic reference frame for the effects of true polar wander [11] allowed determining the paleolatitude of a plate motion chain relative to today’s mantle structure. Using this, new mantle reference frames were recently developed for geodynamic analyses [43,44]. One of these frames shifts the plate motion chain in longitude to optimize the fit between subduction zones in plate reconstructions and remnants of subduction imaged by seismic tomography (‘slab fitting’) [43]. In addition, plate reconstructions have suggested that mantle plumes are generated at specific regions in the deepest mantle that can be imaged by seismic tomography [45–49]. Geological expressions of mantle plumes such as large igneous provinces (LIPS) and kimberlites have thus also been used to provide paleolongitudinal control relative to the mantle, by fitting these to their possible source areas (Fig 1).

On the other hand, methods to calculate the effect of true polar wander have shown that these may be as large as ~23° in the Late Triassic to Early Jurassic [11], a value that was argued to even be an underestimate due to time averaging that is applied in APWP construction [34,50–52]. If one would use a mantle reference frame to determine a paleolatitude for a paleoclimate study, those real latitudinal changes relative to the Earth’s spin axis remain unaccounted for. This may be further illustrated by a recent study of Steinberger and coworkers [53], who showed that Cenozoic true polar wander contributed by as much as 12° to the northward motion of the north Atlantic realm, thereby preconditioning the late Neogene glaciation of Greenland: when studied in a mantle reference frame, this 12° northward motion remains unnoticed.

### Choosing the appropriate reference frame for paleoclimate studies

Paleoclimate studies should in all cases use paleomagnetic data to determine the paleolatitude of deposition of a studied site relative to the Earth’s spin axis. As mentioned above, the paleolatitude inferred for a Paleocene site could be misplaced by as much as 12° (1300 km) relative to the spin axis if one would use the global moving hotspot frame [10] instead of a paleomagnetic reference frame, which may significantly influence the conclusions drawn for paleoclimate reconstructions.

Muttoni and colleagues [50,54] showed that in the Apennines of Italy the Late Triassic-Early Jurassic true polar wander event is associated with a marked facies change from sub-tropical carbonates to tropical radiolarian cherts and back. If one would estimate the paleolatitudes of deposition of these rocks using a mantle reference frame, one would be inclined to explain this result from exogenically-forced climate changes rather than a southward and then northward paleolatitude shift relative to the spin axis.

It is therefore crucial to use the appropriate reference frame when estimating a paleolatitude relative to the Earth’s spin axis. Erroneously using a mantle reference frame for climate studies, no matter how high the quality of that mantle reference frame, may introduce errors in perceived latitude relative to the Earth’s orbit of thousands of kilometers, and tens of degrees of latitude in extreme cases. Paleoclimate studies need to use paleolatitudinal information from paleomagnetic data, and the most accurate results will be obtained when the studied site can be placed in a plate motion chain placed in a global paleomagnetic reference frame defined by the GAPWaP.

## An Online Paleolatitude Calculator: www.paleolatitude.org

**Fig 2:**
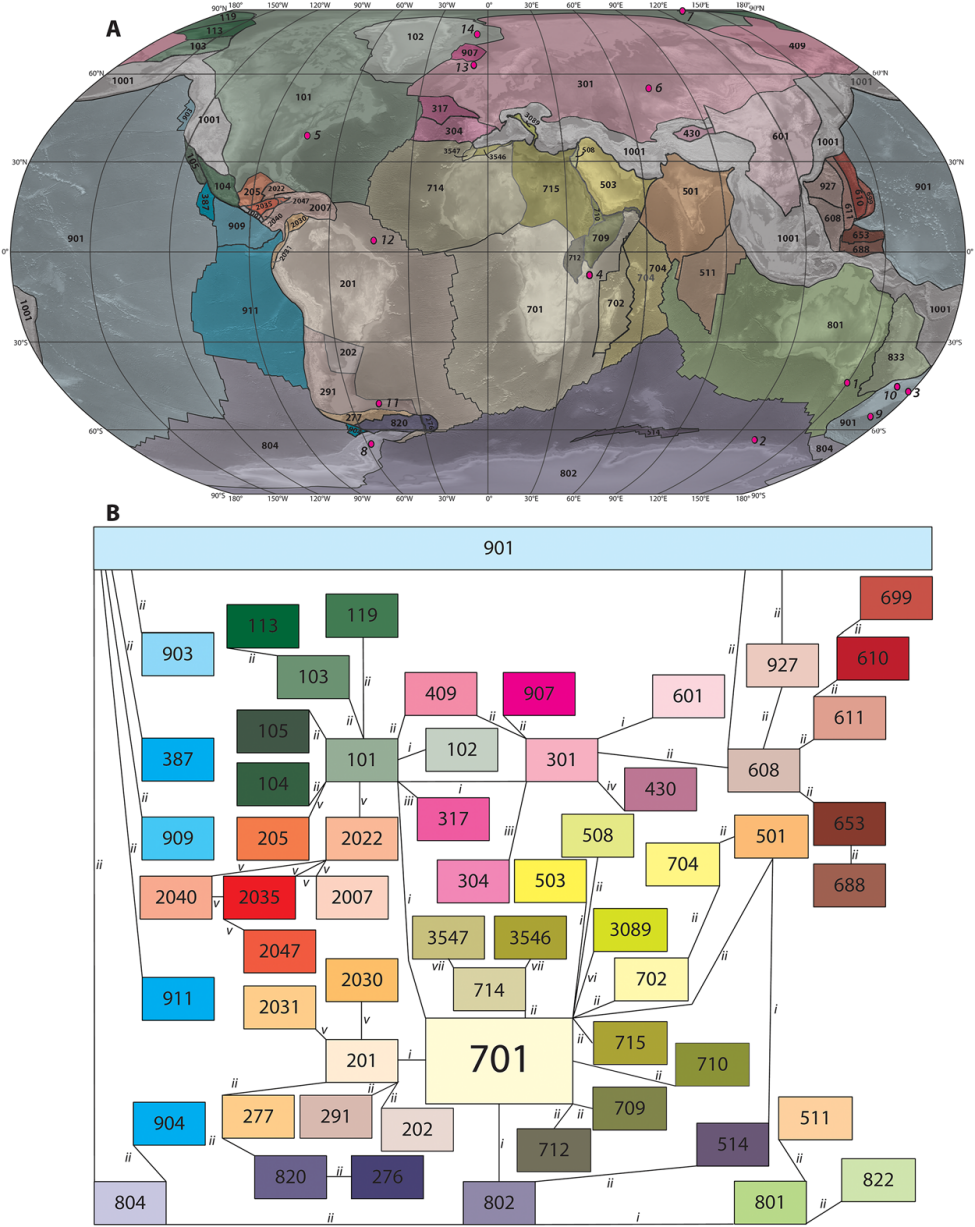
(A) Map with the main lithospheric fragments that have moved independently relative to surrounding fragments in the past 200 Ma. (B) Plate circuit of all fragments shown in A. Rotation parameters relative to South Africa (701) are given for all fragments in Online Appendices 1–3. Names of all elements are given in Table 1. Rotation parameters are taken from (i) ref [20], or ref [18], or ref [34], depending on the reference frame; (ii) ref [14]; (iii) ref [58–60]; (iv) ref [67]; (v) ref [57]; (vi) ref [61]; (vii) ref [62]. Italic number 1–14 indicate the locations of the sites used for a case study on Eocene meridional temperature, see Fig 5, and Table 2.

To unify the datasets that are used in paleoclimate studies, we have developed an online tool to calculate the paleolatitude of a given site, where possible for the last 200 Ma. This calculator can be found on the website www.paleolatitude.org. The version of this website that is described in this paper is version 1.0. We will update this website in the future, for instance expanding it farther back in time, including new GAPWaP’s, or incorporating updated plate reconstructions, and when we do, add a new version of the paper (1.1, 1.2, etc), with specifications on the nature of the update clearly indicated on the site. When citing this paper, and this website, the user is advised to specify the used version of the online calculator. The 1.0 calculator contains options to calculate paleolatitudes using three different paleomagnetic reference frames. The default frame is the GAPWaP of Torsvik and colleagues [20] (S4 Table). These authors used paleomagnetic data from a plate motion chain that contained Eurasia, Greenland, North America, South America, East Antarctica, India, Australia, and Africa, and we have adopted the same (Euler) rotation parameters for the relative motions between these plates that were used to develop the GAPWaP. To this basic plate motion chain, we added plates, such as the Pacific plate, and lithospheric fragments that were part of different plates in the last 200 Ma (Fig 2). An example of the latter is the Seychelles fragment (704, Fig 2), which has never been an independent plate, but has been part of both India and Africa (e.g., [55,56]) and therefore has its own unique set of rotation parameters. In addition, the user can choose the older, but widely used paleomagnetic reference frame of Besse and Courtillot [18], which is broadly similar, and the one of Kent and Irving [34], which only contains paleomagnetic poles from sedimentary rocks if those were explicitly corrected for a paleomagnetic artifact known as ‘inclination shallowing’ that results from compaction of sedimentary rocks (S4 Table). This frame therefore is based on a much smaller dataset, but was argued by the authors to be more accurate than frames based on large, but uncorrected data sets. Kent and Irving [34] built their APWP inspired by the finding of paleomagnetic data in the Jurassic that showed significant differences with other global apparent polar wander paths e.g., [50,52], probably as a result of the 20 Myr sliding window used in APWP calculations that smoothens a sharp cusp on a timescale shorter than 20 Myr. This sharp Jurassic cusp is believed be caused by true polar wander [52,54]. The Kent and Irving [34] reference frame was not defined for the period between 0 and 50 Ma, and is only applied before that time. The reference frames of Besse and Courtillot [18] and Kent and Irving [34] used rotation parameters for the main continents as well as time scales that are subtly different from the ones used by Torsvik and colleagues [20]. The paleolatitude calculator uses the specific rotation parameters and ages that were used by the authors of each paleomagnetic reference frame.

The plate circuits defined in each frame are then expanded with reconstructions of areas from which no paleomagnetic data were used to build the reference frame. Rotation parameters for the (circum-) Pacific Realm were adopted from the global *GPlates* [13] reconstruction of Seton and colleagues [14]. The plates of the Pacific can only be incorporated in our plate motion chain starting from Late Cretaceous times, because prior to that the Pacific plates did not share a passive margin with any of the surrounding continents [32]. We incorporated a simplification of a recent restoration of the Caribbean region [57] as well as recent restorations of the Bay of Biscay and Iberia [58–60], Adria [61] and NW Africa [62] (Fig 2; Table 1). The resulting circuit contains 61 fragments (Fig 2; Table 1), for which rotation parameters relative to South Africa are given in the online Appendices 1–3 (one per reference frame). Many sedimentary sections studied on land are exposed in fold-thrust belts of, e.g., the Alpine-Himalayan mountain belt or western North America. We have not included these complexly deformed areas in this tool at this moment; reconstruction of these zones are underway, and have considerably larger error margins. Paleomagnetic and kinematic reconstructions of western North America [34,63–65], the Tethyan realm [62,66–74]) and the southwest Pacific [75,76] are anticipated to be included in the paleolatitude calculator in the future. Because most paleoclimate studies, including those based on ocean drilling, are generally carried out in tectonically relatively inactive regions, notably in continental and oceanic basins and at passive margins, we restrict ourselves now to the stable plate interiors.

**Table 1:** List of codes and associated polygons representing plates or plate fragments used to build the global plate motion chain of Fig 2.

Plate ID	Plate name	Plate name	Plate ID
101	North America	Africa	701
102	Greenland	Amerasia Basin	119
103	North Slope Alaska	Arabia	503
104	Mexican terranes	Australia	801
105	Baja California	Baja California	105
113	Northwind Ridge	Capricorn	511
119	Amerasia Basin	Caribbean	2007
201	South America	China Blocks & Amuria	601
205	Yucatan	Chortis Block	2035
276	Sandwich	Cocos	909
277	North Scotia	Cuba segment	2022
301	Eurasia	East Antarctica	802
304	Iberia	East Pareve Vela	610
317	Porcupine	Elan Bank	514
387	Riveria	Eurasia	301
409	Northeast Siberia	Greater Panama	2031
430	Tarim	Greenland	102
501	India	Iberia	304
503	Arabia	India	501
511	Capricorn	Jan Mayen	907
514	Elan Bank	Juan de Fuca	903
601	China Blocks & Amuria	Lord Howe Rise	833
608	South Phillipine Sea	Madagascar	702
610	East Pareve Vela	Maracaibo Block	2030
611	North Parece Vela	Marianas forearc	699
653	North Caroline Sea	Mexican terranes	104
688	Southwest Caroline Sea	Mobile belt—unconstrained	1001
699	Marianas forearc	Nazca	911
701	Africa	North America	101
702	Madagascar	North Caroline Sea	653
704	Seychelles	North Nicaraguan Rise	2047
714	NW Africa	North Parece Vela	611
801	Australia	North Philippine Sea	927
802	East Antarctica	North Scotia	277
804	West Antarctica	North Slope Alaska	103
820	South Scotia	Northeast Siberia	409
833	Lord Howe Rise	Northwind Ridge	113
901	Pacific	NW Africa	714
903	Juan de Fuca	Pacific	901
904	Phoenix	Phoenix	904
907	Jan Mayen	Porcupine	317
909	Cocos	Riberia	387
911	Nazca	Sandwich	276
927	North Philippine Sea	Seychelles	704
1001	Mobile belt—unconstrained	Siuna Terrane	2040
2007	Caribbean	South America	201
2022	Cuba segment	South Phillipine Sea	608
2030	Maracaibo Block	South Scotia	820
2031	Greater Panama	Southwest Caroline Sea	688
2035	Chortis Block	Tarim	430
2040	Siuna Terrane	West Antarctica	804
2047	North Nicaraguan Rise	Yucatan	205

Euler poles for all polygons relative to South Africa are given in S1, S2, and S3 Tables.

**Fig 3:**
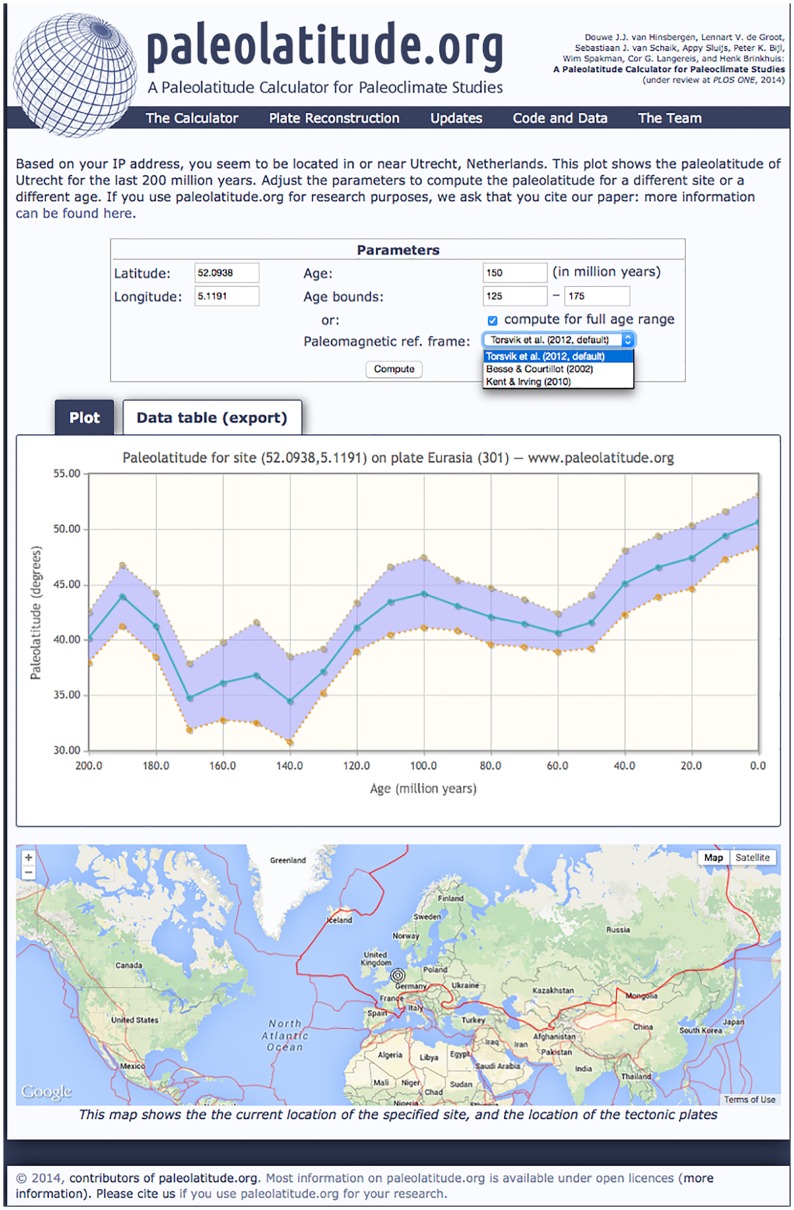
Screen shot of www.paleolatitude.org. In the top panel, site latitude and longitude is required (in decimal degrees), the age of the site, and the lower and upper age bounds. Center panel shows the paleolatitude with error bars in the period between the upper and lower bounds. The option ‘compute for full age range’ will calculate a paleolatitude curve using the full age range of the plate (fragment) on which the chosen site is located, which will appear in the center panel. Second tab in the center panel will provide a table with an output file for paleolatitudes with error bounds in the chosen time interval. Note that at the location of this site (e.g., close to a mid-ocean ridge) the plate may not have existed throughout this entire history. The bottom panel shows the location of the site on the modern topographic world map, as well as the plate (fragment) on which the site is located. Reprinted from www.paleolatitude.org under a CC BY license, with permission from S.J. van Schaik, original copyright 2014.

The paleolatitude calculator uses the following approach. The GAPWaP is defined in a (South) African coordinate frame [20]. The online paleolatitude calculator only requires present-day geographic coordinates as input, and automatically assigns this to the appropriate plate or plate fragment (Fig 3). The apparent location of the paleomagnetic north pole for this plate fragment through time is then derived by rotating the GAPWaP into the coordinates of this fragment using the Euler rotation parameters given in the online S1, S2, and S3 Tables.

The paleolatitude of a specific location and for a given age on the selected plate is then calculated as follows. The GAPWaP provides the apparent position of the paleomagnetic pole for the chosen plate, with a confidence region given as A95 [20]. At the chosen position on that plate, the calculated paleomagnetic pole corresponds to a paleomagnetic inclination and its error I ± Δ*I*_x_ from which the paleolatitude and its error is computed using Eq (1). The dipole equation causes the error in paleolatitude to be asymmetrical; the error margin is calculated via

$$tan(I+\Delta I_{x})\;=\;2\;tan\lambda_{hi}$$ (2a)

$$tan(I-\Delta I_{x})=2\;tan\lambda_{lo}$$ (2b)

We do not incorporate additional errors, e.g. associated with the plate reconstruction. These are typically smaller than paleomagnetic errors, but are not quantified everywhere. The error bars given by the paleolatitude calculator should therefore be considered as minimum values. Our paleolatitude calculator linearly interpolates latitudes and confidence intervals within the 10 Myr intervals of the GAPWaP.

We incorporate the effect of age uncertainty on paleolatitude uncertainty by calculating the average paleolatitude, and its upper and lower bounds for each 10 Myr step within the time interval specified. The paleolatitude returned by the calculator is obtained from linear interpolation at the specified age, while the upper and lower bounds for the paleolatitude are the maximum and minimum occurring values within the time interval specified. The mathematical details of the routine are fully described in the S1 Text.

## Case Study: Eocene Meridional Sea Surface Temperature Gradients

**Fig 4:**
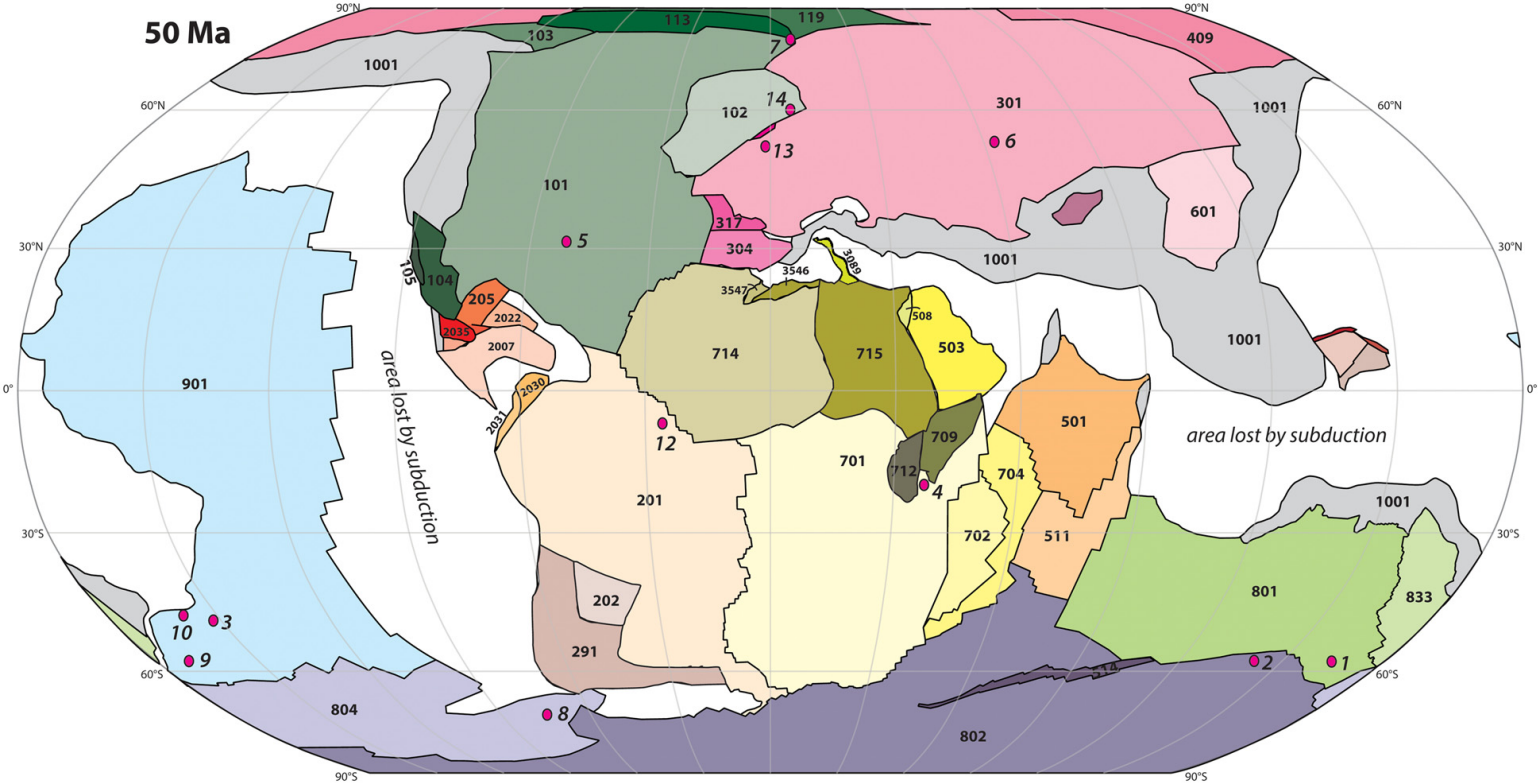
Plate reconstruction at 50 Ma, around the moment of the Early Eocene Climate Optimum, with the sites used for sea surface temperature estimates in Figs 1 and 5. Reconstruction made in *GPlates* from Seton and colleagues [14], with modifications as indicated in the main text, placed in the paleomagnetic reference frame of Torsvik and colleagues [20]. Absolute paleolongitude of the global plate motion chain is unconstrained, and irrelevant for paleoclimate reconstructions. Meridians are spaced with 30 degree intervals. Italic numbers 1–14 indicate the reconstructed locations of the sites used for a case study on Eocene meridional temperature, see Fig 5, and Table 2.

The Eocene is an important time interval for paleoclimatology, because Eocene atmospheric CO_2_ concentrations may have been similar to those of our near future assuming unabated carbon emissions [77]. In light of this, a large body of paleoclimate research has been undertaken in recent years to reconstruct Eocene climate. One notably interesting result from this research is that there apparently were flat meridional paleotemperature gradients in the Eocene according to a compilation of Bijl et al [2], who used paleolatitudes as reported in the studies that were compiled, whereby the source of these reported paleolatitudes are generally not specified in detail. Particularly the high latitudes were much (>20°C) warmer than today, while mid-and low latitude paleotemperatures were only moderately warmer (Fig 4, [2]). Although some cooling occurred during the middle Eocene, the Southern Ocean sea surface temperatures (SSTs) still stood out as extraordinarily warm during this time period. Numerical models are used to assess the radiative forcing required to produce these temperatures. However, to match the high latitude temperature reconstructions, the current generation of fully coupled models require radiative forcing so strong that mid-latitude and equatorial temperatures should have been much higher than existing data suggest (e.g., [3]).

We use this case study to illustrate the use of the paleolatitude calculator: we test whether the detailed plate model (Fig 2) and the latest paleomagnetic reference frame [20] leads to corrections in the paleolatitudes originally determined for the data points, and we determine lower-bound uncertainties of the corrected paleolatitudes. The southern high latitude SST data come from the SW Pacific, SE Indian and SW Atlantic Oceans, specifically from the East Tasman Plateau [2,12], the Wilkes Land Margin of East Antarctica [12], New Zealand [78,79] and Seymour Island, Antarctic Peninsula [80] (Figs 2 and 4, Table 2).

**Table 2:** Locations, ages, sea surface temperature calculations with standard deviation, and paleolatitude values for sites used to calculate meridional temperature profiles for time slices in the Early (55–48 Ma) and Middle Eocene (45–39 Ma).

													Torsvik et al [20]			Besse and Courtillot [18]			Kent and Irving [34]	
Site number	Site name	Plate ID	Present-day latitude (°)	Present-day longitude (°)	Age (Ma)	Age (min) (Ma)	Age (max) (Ma)	Published paleolatitude (°)	SST (TEX86H) (°C)	1σ SST (°C)	Source	Paleolatitude (°)	Northern limit paleolatitude (°)	Southern limit paleolatitude (°)	Paleolatitude (°)	Northern limit paleolatitude (°)	Southern limit paleolatitude (°)	Paleolatitude (°)	Northern limit paleolatitude (°)	Southern limit paleolatitude (°)
*early Eocene*																				
1	ODP Site 1172	801 (Australia)	-43.96	149.93	51.5	48.0	55.0	-65	28.9	2.4	[2]	-57.9	-52.8	-62.8	-59.1	-52.7	-65.9	-60.5	-50.6	-72.5
2	IODP Site U1356	802 (East Antarctica)	-63.01	135.00	51.5	48.0	55.0	-65	32.0	0.7	[12]	-57.7	-52.6	-62.7	-59.5	-53.1	-66.3	-59.4	-49.8	-71.1
3	Mid-waipara	901 (Pacific)	-43.16	172.77	51.5	48.0	55.0	-60	31.0	1.0	[78, 79]	-48.1	-44.1	-52.0	-48.8	-44.0	-53.9	-51.1	-43.9	-59.8
4	Tanzania	701 (South Africa)	-7.77	39.06	51.5	48.0	55.0	-19	33.0	1.7	[81]	-22.0	-19.6	-24.4	-21.1	-19.0	-23.3	-19.7	-17.1	-22.5
5	New Jersey	101 (North America)	39.61	-74.44	51.5	48.0	55.0	35	30.5	2.0	[82]	34.1	36.2	31.9	36.6	39.4	33.7	35.3	31.3	39.9
6	Siberie	301 (Eurasia)	53.50	73.50	51.5	48.0	55.0	58	23.2	2.1	[83]	51.8	56.6	47.9	43.8	48.3	39.9	53.5	62.3	46.0
7	Arctic Lomonosov Ridge	101 (North America)	87.87	136.19	51.5	48.0	55.0	85	21.4	2.5	[83, 84, 85]	78.2	88.3	68.6	78.6	90.0	67.4	80.3	90.0	61.5
*middle Eocene*																				
8	Seymour Island	804 (West Antarctica)	-64.28	-56.75	42.0	39.0	45.0	-67	20.9	2.1	[80]	-70.4	-62.7	-78.9	-68.3	-60.1	-77.0			
1	ODP Site 1172	801 (Australia)	-43.96	149.93	42.0	39.0	45.0	-65	24.8	1.4	[2]	-57.7	-52.8	-62.9	-57.1	-51.3	-63.6			
9	DSDP Site 277	901 (Pacific)	-52.22	166.19	42.0	39.0	45.0	-62	25.2	2.5	[79]	-55.0	-50.7	-59.9	-54.3	-49.2	-60.0			
10	Hampden beach, NZ	901 (Pacific)	-43.09	172.77	42.0	39.0	45.0	-60	24.3	0.4	[86]	-47.6	-44.3	-51.2	-46.4	-42.4	-50.7			
11	DSDP Site 511	291 (Colorado-San Jorge)	-51.00	-46.97	42.0	39.0	45.0	-55	27.0	2.5	[87]	-59.4	-54.0	-64.8	-58.6	-52.9	-64.9			
4	Tanzania	701 (South Africa)	-7.77	39.06	42.0	39.0	45.0	-19	34.0	0.1	[88]	-17.3	-14.8	-19.9	-18.7	-16.7	-20.7			
12	ODP Site 925	201 (South America)	4.20	-43.49	42.0	39.0	45.0	-2	33.0	2.5	[89]	-4.2	-2.9	-5.4	-3.4	-2.4	-4.4			
13	DSDP Site 336	301 (Eurasia)	63.35	-7.79	42.0	39.0	45.0	60	23.0	4.0	[90]	54.7	60.0	49.8	49.7	55.0	45.0			
14	ODP Site 913	102 (Greenland)	75.85	-6.95	42.0	39.0	45.0	65	25.0	4.0	[90]	64.5	72.8	57.3	63.9	72.4	56.4			
7	Arctic Lomonosov Ridge	101 (North America)	87.87	136.19	42.0	39.0	45.0	85	11.3	1.9	[91]	81.2	90.0	70.2	80.2	90.0	68.2			

Published paleolatitudes refer to values published by the original authors. Values in column paleolatitude.org and error are calculated using methods presented in this paper.

Recently, paleotemperature data from Seymour Island, Antarctic Peninsula, South Atlantic Ocean were reported that suggested much (~5°C) cooler middle Eocene paleotemperatures than those from time-equivalent sites in the SW Pacific Ocean [80].

Paleolatitude reconstructions used by these papers suggested that these sites were all located at about the same paleolatitude of ~65°S [2,80]. Paleoceanographic reconstructions suggested that both sites were under Antarctic-derived surface current influence [92,93], and therefore, similar paleo-SSTs were expected [80].

**Fig 5:**
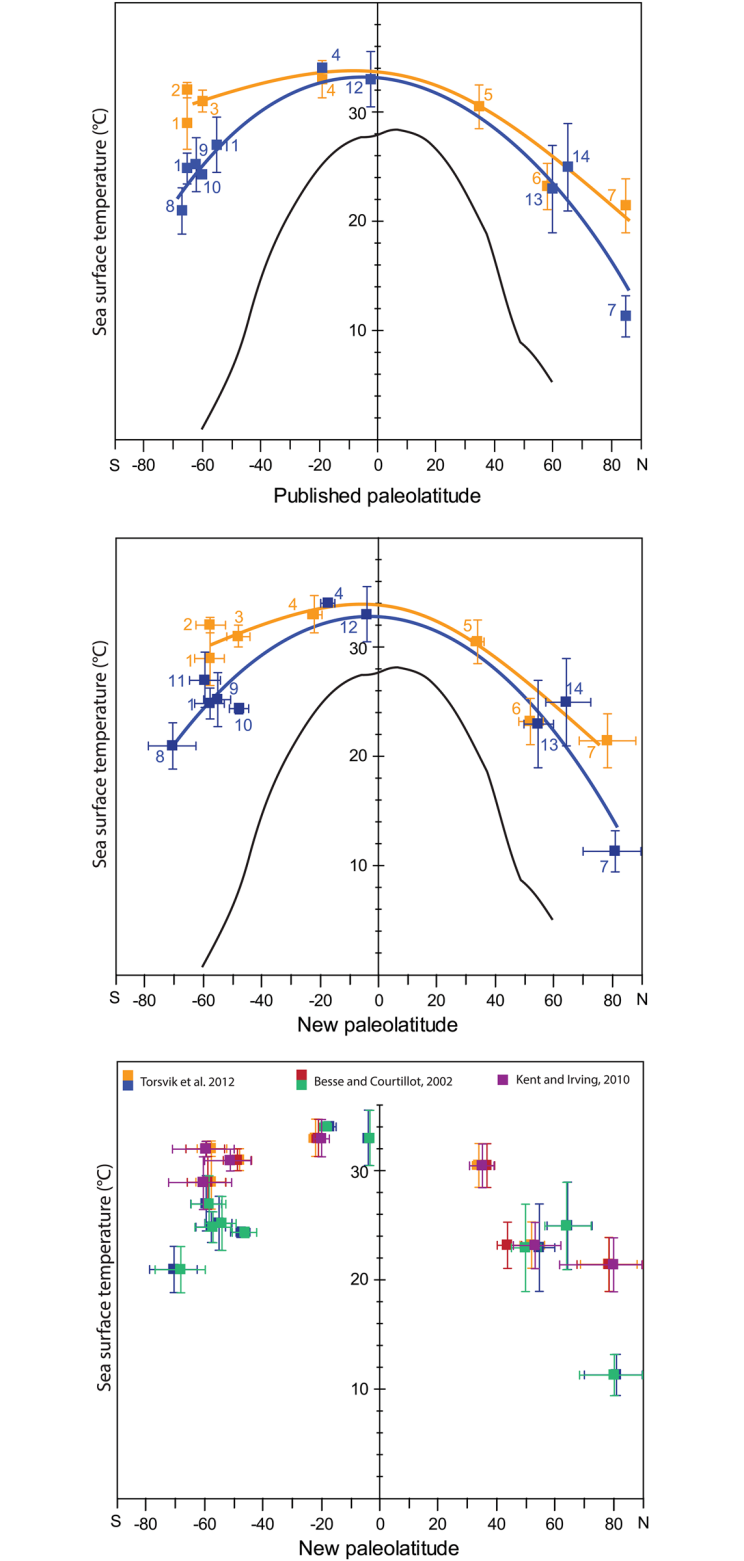
Latitudinal sea surface temperature (SST) gradients for the early- (orange) and middle (blue) Eocene (modified from Bijl and colleagues [2]). See text for full derivation of data. TEX_86_-derived SSTs (squares) were recalibrated to TEX_86_-H following Kim and colleagues [94]. (A) with paleolatitudes as published by Bijl and colleagues [2] and references therein, (B) with paleolatitudes and error bars using the paleolatitude calculator provided with this paper (www.paleolatitude.org), in the default reference frame of Torsvik et al. [20]. (C) comparison of paleolatitudes of the same paleotemperature data using paleomagnetic reference frames of Torsvik et al. [20], Besse and Courtillot [18] and Kent and Irving [34]. For data, see Table 2, for present-day locations see Fig 2, for reconstructed locations at 50 Ma see Fig 4.

Our paleolatitude calculator introduces error bars on the paleolatitude estimates, which at high latitudes may amount to ~15° in the Eocene, and determines significant shifts in the paleolatitude of some sites (Fig 5). For instance, the most recent IPCC report [1] places the continents Africa, Europe, North and South America more than 10° farther north than predicted paleomagnetically. In addition, when we recalculate the paleolatitudes of the sites that created the enigma on the middle Eocene paleotemperatures from the South Atlantic and southwest Pacific Ocean, it becomes clear that the site on Seymour island, which gave much cooler paleotemperatures than time-equivalent sites in the South Pacific realm, was located ~12° (~1300 km) farther south than the Pacific sites relative to the Earth’s spin axis (Fig 5, Table 2). This latitudinal separation allows for a steeper SST gradient in the middle Eocene, which makes the numerical modeling studies more compatible with observations. Notably, the apparent improvement in paleotemperature gradient reconstructions is consistent for the three paleomagnetic reference frames of Torsvik et al. [20], Besse and Courtillot [18] and Kent and Irving [34]: differences in paleolatitude reconstructions are within error of each other (Fig 5c). The largest difference between frames is noticeable around the locations of the paleomagnetic poles, in Siberia. A site close to the Russian-Kazakhstan border [83] has Early Eocene paleolatitudes that are ~10 degrees different between the Torsvik et al. [20] and Besse and Courtillot [18] frames (Fig 5c). Irrespective of the reference frame applied, our case study illustrates the importance of reconstructing accurate paleolatitudes in paleoclimate studies, and the straightforward applicability of the paleolatitude calculator in such studies.

## Summary and Conclusions

In this paper, we summarize how the latest state of the art of global plate reconstructions and paleomagnetic reference frames provide the appropriate boundary condition for paleoclimate studies. Widely used so-called ‘mantle reference frames’ that have been developed for geodynamic research purposes cannot constrain (or were specifically corrected for) true polar wander. Because true polar wander does change the latitude of the Earth’s surface relative to the Earth’s spin axis, it is essential for paleoclimate studies to use a reference frame that includes the effects of true polar wander, i.e. a paleomagnetic reference frame. Using a mantle reference frame for paleoclimate studies could introduce a bias of more than 20° in the last 200 Myr.

Using the latest state-of-the-art of global plate reconstructions, and three recent global apparent polar wander paths, we built a user-friendly, online available paleolatitude calculator (www.paleolatitude.org) for the last 200 Myr, tailored for paleoclimate (or paleomangnetic) studies. We demonstrate the applicability and use of this tool through a case study on Eocene meridional sea surface temperature profiles: we show how enigmatically strongly varying Southern Ocean sea surface temperatures may be explained by a much larger paleolatitudinal spread of sample sites than previously appreciated.

## Supporting Information

S1 Table Total reconstruction Euler poles relative to South Africa for all elements in the plate circuit of Fig 2 of the main paper, using the rotation parameters for Africa, South America, North America, Greenland, Eurasia, Australia, East Antarctica, Madagascar, and India of Torsvik and colleagues [20], and for the remaining elements as detailed in the caption of Fig 2 in the main text. (XLSX)

S2 Table Total reconstruction Euler poles relative to South Africa for all elements in the plate circuit of Fig 2 of the main paper, using the rotation parameters for Africa, South America, North America, Greenland, Eurasia, Australia, East Antarctica, Madagascar, and India of Besse and Courtillot [18], and for the remaining elements as detailed in the caption of Fig 2 in the main text. (XLSX)

S3 Table Total reconstruction Euler poles relative to South Africa for all elements in the plate circuit of Fig 2 of the main paper, using the rotation parameters for Africa, South America, North America, Greenland, Eurasia, Australia, East Antarctica, Madagascar, and India of Kent and Irving [34], and for the remaining elements as detailed in the caption of Fig 2 in the main text. (XLSX)

S4 Table Apparent Polar Wander Paths in South African coordinates of Torsvik and colleagues [20], Besse and Courtillot [18], and Kent and Irving [34]. (XLSX)

S1 Text Mathematical workflow behind the paleolatitude calculation carried out by the paleolatitude.org website. (PDF)
