# Supplementary material for: A Paleolatitude Calculator for Paleoclimate Studies
Source: PLoS One. 2015 Jun 10;10(6):e0126946. doi: 10.1371/journal.pone.0126946 (PMC4462584; doi:10.1371/journal.pone.0126946)
Supplement: S1 Text — (PDF) [file pone.0126946.s005.pdf]

Supplementary information: the routine

Variables

λ_s	latitude of the site	input
φ_s	longitude of the site	input
age	age	input
age_{max}	upper bound of age	input
age_{min}	lower bound of age	input
age_{pm}	Gaussian error around age	input
plate	the applicable geological plate	
λ_p	latitude of the reference pole	
φ_p	longitude of the reference pole	
θ_p	colatitude of the reference pole	
A_{95}	95% confidence interval of reference pole	
λ_E	latitude of the Euler pole	
φ_E	longitude of the Euler pole	
θ_E	colatitude of the Euler pole	
Ω	rotation around the Euler pole	
r_E	rotation axis	
$\boldsymbol{L}$	coordinate transformation matrix	
$\lambda_{,p,rot}$	latitude of the rotated reference pole	
$\varphi_{p,rot}$	longitude of the rotated reference pole	
$\theta_{p,rot}$	colatitude of the rotated reference pole	
Λ	paleolatitude of the site	output
I	inclination of the geomagnetic field at paleolatitude	
Δ_I	uncertainty in inclination	
Λ_{max}	upper bound of paleolatitude	output
Λ_{min}	lower bound of paleolatitude	output

Workflow

1. Input: λ_s, φ_s, age, and (age_{max} & age_{min}, or age_{pm})

2. if age_{max}, age_{min} = 0, calculate them from age_{pm}

$$\text{age}_{max} = \text{age} + \text{age}_{pm}$$
$$\text{age}_{min} = \text{age} - \text{age}_{pm}$$

3. Determine age levels needed to obtain a proper error estimate. The reference poles are determined for each 10 million years. The relevant ages are therefore determined as follows:

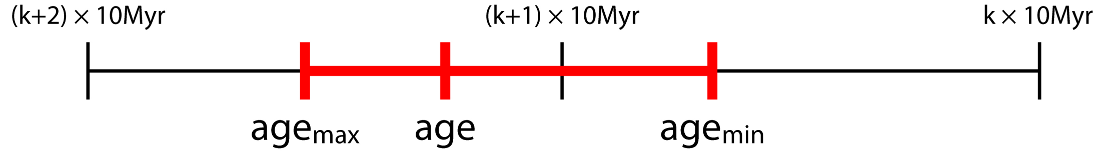

Here Λ, Λ_{max}, and Λ_{min} should be calculated for k×10Myr, (k+1)×10Myr, and (k+2)×10Myr. For these ages the following workflow is to be followed:

a. Select tectonic plate based on GIS
    i. λ_s, φ_s, ➔ plate (select plate-ID from GIS-file)

b. Select Euler pole and reference pole from table
    i. plate, age ➔ λ_E, φ_E, Ω
    ii. age ➔ λ_p, φ_p, A95 (apparent polar wander paths)

c. Calculate the colatitudes of the Euler pole and reference pole

$$\theta_E = 90 - \lambda_E$$
$$\theta_p = 90 - \lambda_p$$

d. Rotate the reference pole around the Euler pole by Ω
    i. Define the x-, y-, z-unit vectors for the coordinate transformation matrix ***L***

$$\hat{x} = \begin{bmatrix}1\\0\\0\end{bmatrix} \qquad \hat{y} = \begin{bmatrix}0\\1\\0\end{bmatrix} \qquad \hat{z} = \begin{bmatrix}0\\0\\1\end{bmatrix}$$

    ii. Define the θ_E-, φ_E-, r_E-unit vectors for matrix ***L***

$$\hat{\theta}_E = \begin{bmatrix}\cos\varphi_E \cos\theta_E\\ \sin\varphi_E \cos\theta_E\\ -\sin\theta_E\end{bmatrix} \qquad \hat{\varphi}_E = \begin{bmatrix}-\sin\varphi_E\\ \cos\varphi_E\\ 0\end{bmatrix} \qquad \hat{r}_E = \begin{bmatrix}\cos\varphi_E \sin\theta_E\\ \sin\varphi_E \sin\theta_E\\ \cos\theta_E\end{bmatrix}$$

    iii. Define coordinate transformation matrix L

$$\boldsymbol{L} = \begin{bmatrix}\hat{\theta}_E \cdot \hat{x} & \hat{\varphi}_E \cdot \hat{x} & \hat{r}_E \cdot \hat{x}\\ \hat{\theta}_E \cdot \hat{y} & \hat{\varphi}_E \cdot \hat{y} & \hat{r}_E \cdot \hat{y}\\ \hat{\theta}_E \cdot \hat{z} & \hat{\varphi}_E \cdot \hat{z} & \hat{r}_E \cdot \hat{z}\end{bmatrix}$$

    iv. Convert reference pole to Cartesian coordinates

$$\begin{bmatrix}\cos\varphi_p \sin\theta_p\\ \sin\varphi_p \sin\theta_p\\ \cos\theta_p\end{bmatrix} = \begin{bmatrix}x_p\\ y_p\\ z_p\end{bmatrix}$$

    v. Perform Euler pole rotation (backwards, hence $-\Omega$)

$$\boldsymbol{L}\begin{bmatrix}\cos -\Omega & -\sin -\Omega & 0\\ \sin -\Omega & \cos -\Omega & 0\\ 0 & 0 & 1\end{bmatrix}\boldsymbol{L}^T\begin{bmatrix}x_p\\ y_p\\ z_p\end{bmatrix} = \begin{bmatrix}x_{p,rot}\\ y_{p,rot}\\ z_{p,rot}\end{bmatrix}$$

    vi. Convert the rotated pole to spherical coordinates
    IF $x_{p,rot} < 0$ THEN:

$$\begin{bmatrix}\varphi_{p,rot}\\ \theta_{p,rot}\end{bmatrix} = \begin{bmatrix}\tan^{-1}\dfrac{y_{p,rot}}{x_{p,rot}} + 180\\ \cos^{-1} z_{p,rot}\end{bmatrix}$$

IF $x_{p,rot} \geq 0$ AND $y_{p,rot} \geq 0$ THEN:

$$\begin{bmatrix} \varphi_{p,rot} \\ \theta_{p,rot} \end{bmatrix} = \begin{bmatrix} \tan^{-1} \frac{y_{p,rot}}{x_{p,rot}} \\ \cos^{-1} z_{p,rot} \end{bmatrix}$$

IF $x_{p,rot} \geq 0$ AND $y_{p,rot} < 0$ THEN:

$$\begin{bmatrix} \varphi_{p,rot} \\ \theta_{p,rot} \end{bmatrix} = \begin{bmatrix} \tan^{-1} \frac{y_{p,rot}}{x_{p,rot}} + 360 \\ \cos^{-1} z_{p,rot} \end{bmatrix}$$

vii. Calculate latitude of rotated reference pole

$$\lambda_{p,rot} = 90 - \theta_{p,rot}$$

e. Calculate the paleolatitude

$$\Lambda = \tan^{-1} \frac{\sin \lambda_{p,rot} \sin \lambda_s + \cos \lambda_{p,rot} \cos \lambda_s \cos(\varphi_{p,rot} - \varphi_s)}{\sqrt{1 - (\sin \lambda_{p,rot} \sin \lambda_s + \cos \lambda_{p,rot} \cos \lambda_s \cos(\varphi_{p,rot} - \varphi_s))^2}}$$

f. Determine the uncertainty in the paleolatitude

$$\Delta_I = A_{95} \left(\frac{2}{1 + 3\cos^2(90 - \Lambda)} \right)$$

g. Calculate upper and lower bounds for the paleolatitude

i. Determine the inclination of the geomagnetic field at the paleolatitude

$$I = \tan^{-1} \frac{2 \times \left(\sin \lambda_{p,rot} \sin \lambda_s + \cos \lambda_{p,rot} \cos \lambda_s \cos\left(\varphi_{p,rot} - \varphi_s\right)\right)}{\sqrt{1 - \left(\sin \lambda_{p,rot} \sin \lambda_s + \cos \lambda_{p,rot} \cos \lambda_s \cos\left(\varphi_{p,rot} - \varphi_s\right)\right)^2}}$$

ii. Calculate the upper and lower bounds for the paleolatitude

$$\Lambda_{min} = \tan^{-1}(0.5 \tan(I - \Delta_I))$$
$$\Lambda_{max} = \tan^{-1}(0.5 \tan(I + \Delta_I))$$

4. Interpolate Λ for age

$$\Lambda = (\text{age} - (k + 2) \times 10\text{Myr}) \left(\frac{\Lambda_{k+1} - \Lambda_{k+2}}{10\text{Myr}} \right) + \Lambda_{k+2}$$

5. Interpolate Λ_{max}, and Λ_{min} for age_{max} and age_{min}

$$\Lambda_{age_{max}} = (\text{age}_{max} - (k + 2) \times 10\text{Myr}) \left(\frac{\Lambda_{max,k+1} - \Lambda_{max,k+2}}{10\text{Myr}} \right) + \Lambda_{max,k+2}$$

etc, etc…

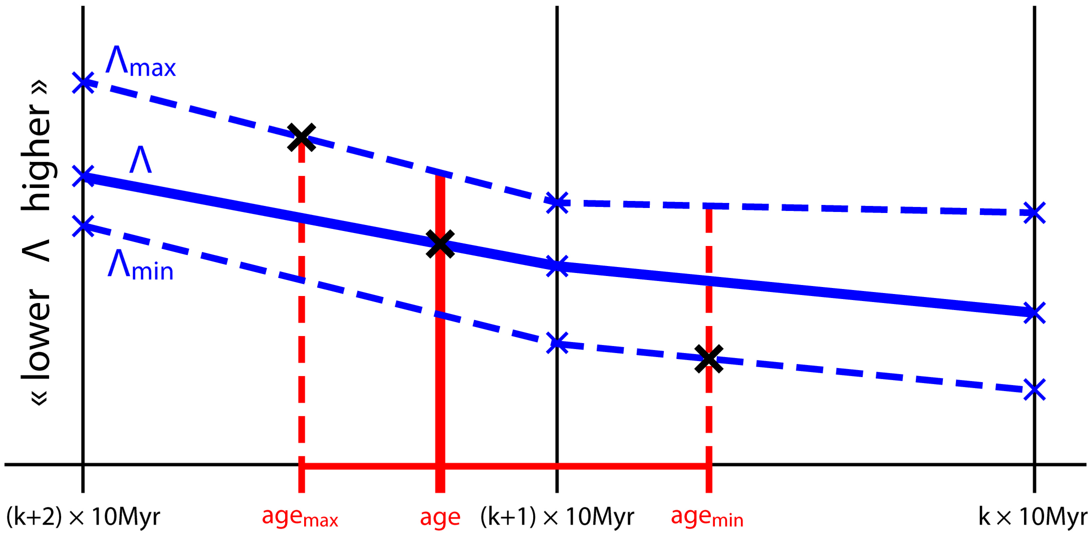

6. Select the maximum occurring value of Λ_{max}, and the minimum occurring value of Λ_{min} (the black crosses in the figure above)

$$\Lambda_{max} = \max\left(\Lambda_{max,age_{max}}, \Lambda_{max,k+1}, \Lambda_{max,age_{min}}\right)$$
$$\Lambda_{min} = \min\left(\Lambda_{min,age_{max}}, \Lambda_{min,k+1}, \Lambda_{min,age_{min}}\right)$$

7. Output: λ_s, θ_s, plate, age, age_{min}, age_{max}, Λ, Λ_{min}, Λ_{max}
